# Developing personal attributes of professionalism during clinical rotations: views of final year bachelor of clinical medical practice students

**DOI:** 10.1186/1472-6920-14-146

**Published:** 2014-07-16

**Authors:** Nontsikelelo Mapukata-Sondzaba, Ames Dhai, Norma Tsotsi, Eleanor Ross

**Affiliations:** 1 Division of Rural Health, Faculty of Health Sciences, University of the Witwatersrand, 7 York Road, Parktown, Johannesburg 2193, South Africa; 2 Steve Biko Centre for Bioethics, Faculty of Health Sciences, University of the Witwatersrand, Johannesburg, South Africa; 3 Centre for Social Development in Africa, University of Johannesburg, Johannesburg, South Africa

**Keywords:** Professionalism, Personal attributes, Health sciences students, Clinical rotations

## Abstract

**Background:**

Medical professionalism as a set of behaviours that transcends personal values, beliefs and attitudes to incorporate ethical and moral principles is considered a covenant between society and the practice of medicine. The Bachelor of Clinical Medical Practice (BCMP) a three year professional degree was launched at the University of the Witwatersrand in January 2009 in response to a documented shortage of doctors especially in the rural areas of South Africa. The BCMP programme is unique in its offering as it requires a teaching approach that meets the needs of an integrated curriculum, providing for an accelerated transition from the classroom to the patient’s bedside.

**Methods:**

Following five week attachments in designated District Education Campuses, 25 final year BCMP students were required to reflect individually on the covenant that exists between society and the practice of medicine based on their daily interactions with health care workers and patients for three of the five rotations in a one page document. A retrospective, descriptive case study employed qualitative methods to group emerging themes from 71 portfolios. Ethical clearance was obtained from the Human Research Ethics Committee at the University of the Witwatersrand.

**Results:**

As an outcome of an ethical analysis, the majority of BCMP students reflected on the determinants of accountable and responsible practice (N=54). The commitment to the Oath became significant with a personalised reference to patients ‘*as my patients*’. Students acknowledged professional health care workers (HCWs) who demonstrated commitment to core values of good practice as they recognised the value of constantly reflecting as a skill (n=51). As the students reflected on feeling like ‘*guinea pigs*’ (n=25) migrating through periods of uncertainity to become ‘*teachable learners*’, they made ethical judgements that demonstrated the development of their moral integrity. A few students felt vulnerable in instances where they were pressured into ‘*pushing the line’.*

**Conclusions:**

Through their portfolio narratives, BCMP students showed a willingness to shape their evolving journeys of moral growth and personal development. This study has highlighted as an ongoing challenge the need to identify a process by which professionalism is sustained by HCWs to benefit health sciences students.

## Background

Professionalism in the 21st Century has progressed from a hierarchal practice that was defined by social structures to consider not only the knowledge and skills of the health care providers but also, their attributes and behaviours which must be congruent and meet the expectations of society and the profession [1-3]. Professionalism remains a topical issue among academics, practitioners and professional bodies [4]. This interest is largely driven by the fact that the desired doctor-patient relationship demands that students are taught professionalism and communication, recognizing that patients have rights and that health care providers have corresponding obligations to their patients [5]. Cases of self-reported breaches of professionalism and ethical misconduct by students such as cheating and plagiarism, as well as clinicians who display negative and disruptive behaviours, have been documented as a growing concern [1,3,6,7]. These concerns were further highlighted by results from a longitudinal study undertaken by Papadakis *et al*. [8] where they demonstrated a direct correlation between medical students’ unprofessional behaviour and subsequent disciplinary action by professional bodies. Based on expressed concerns, there would appear to be a need to preserve the honour of the medical profession through teaching, assessment and on-going research on professionalism [6,9]. With that proviso the clinical training environment is identified as a critical component as it is the culture of the organization that fosters the attainment of professionalism [6,10].

The Bachelor of Clinical Practice (BCMP), a three-year professional degree offered by the University of the Witwatersrand (Wits) accepted its first intake of students in January 2009 in response to a documented shortage of doctors, especially in the rural areas of South Africa [11]. The BCMP programme sought to complement the existing traditional six year medical training programme which admits students via two routes – the school leavers and the Graduate Entry Medical Programme (GEMP) [12]. The BCMP programme is unique in its approach as training is based on the district health care model, a system of primary health care comprising of a cluster of clinics, community health centres and a Level 1 hospital that seeks to ensure that quality healthcare is accessible to all [13,14]. Rooted in problem based learning similar to the GEMP curriculum, this degree is based on a rigorous, competency based and standardized curriculum (Additional file 1). As part of an integrated curriculum the BCMP students are exposed to the clinical environment in one of the locally based District Education Campuses (DECs) urban underserved or in rural communities from 1st year. Progressively they spend more time in the designated DECs learning to manage diseases in areas where they had theoretical instruction by consulting patients under the supervision of their clinical tutors. In their third and final year, only the first rotation is spent in medical school. For the remainder of the year, the final year BCMP students rotate through different sites, undertaking learning in different disciplines supervised by university-appointed supervisors as well as other on-site clinicians. At the end of each block the students’ knowledge and understanding of the curriculum is assessed through written exams and Objective Structured Clinical Exams (OSCE). In their final year, students sit for a national exam together with other final year students from two other universities offering the same programme, namely: the University of Pretoria and the Walter Sisulu University [http://www.twinningagainstaids.org/documents/CABoolketFinal_lowres.pdf].

In South Africa, the Health Professions Council of South Africa (HPCSA) has a mandate to ensure that faculties of Health Sciences include in their core curriculum, academic instruction on professional ethics, human rights and medical law. Furthermore, it is the responsibility of the HPCSA to ensure that all education and training programmes are academically rigorous and clinically relevant [15]. Responding to this call by the HPCSA, a course in bioethics was introduced into the BMCP curriculum in order to humanize the education and practice of these professionals. Through the infusion of human values and the humanities in health sciences education, it was hoped to achieve the ideal of training not only of scientifically competent but also “humanistically responsive practitioners” [16]. In meeting the needs of the BCMP students, the three basic actions described by Stern and Papadakis [9] were applied, namely setting expectations (Oath taking); providing experience; and, teaching and evaluation as broad concepts of teaching professionalism. The class was divided into groups to facilitate teamwork and reflective practice. Case reports and clinical vignettes were used to facilitate the process of attaining academic and professional integrity. Group discussions focused on a patient-centred approach described by Mueller [6] and the role of BCMP students as future professionals. Their knowledge and understanding was assessed as part of the integrated curriculum [17].

Upon qualification these middle level health care professionals (HCPs) are known as Clinical Associates. In the US, they are known as Physician Assistants (PAs), while in the rest of Africa they are referred to as Clinical Officers, a cadre of HCPs who substitute for, and/or complement, medical officers [18]. In order to strengthen the training of Clinical Associates, Wits entered into a twinning partnership with Emory University School of Medicine [http://www.twinningagainstaids.org/documents/CABoolketFinal_lowres.pdf]. In terms of the Human Resources for Health (HRH) strategy [19], Clinical Associates have been identified as one of the categories of HCPs who will contribute to the strengthening of health care services in the district in the implementation of the re-engineering of primary health care [13]. It should be noted that in our context it is not unusual for the categories of HCPs to be referred to as health care workers (HCWs) especially in instances where collective responsibility is acknowledged. In this article, personal attributes of professionalism are described as the highest standards to which health sciences students are willing to commit so that they can develop through reflective practice the skills and abilities that include an understanding of ethics and the legal framework, critical evaluation and self-directed learning as described by Klenowski and Carnell [20]. Mueller [6] is of the view that these skills enable students to be in a position to integrate acquired theoretical knowledge with professional experiences and promote patient autonomy, social justice and primacy of welfare. In this study, personal attributes were evaluated in terms of accountability and responsibility, critical reflection linked to professional development, as well as personal growth.

## Methods

In conducting the study, the research question sought to establish if current teaching and assessment strategies as evidenced through exhibited personal attributes adequately prepared BCMP students to be reflective practitioners. This paper reflects on views expressed by final year BCMP students in developing personal attributes of professionalism by assessing their ability to integrate theoretical knowledge with clinical experience and function as accountable, responsible and critically reflective practitioners.

Following five week attachments in purposely selected clinical departments in designated DECs, all 25 final year BCMP students were required to reflect on the covenant that exists between society and the practice of medicine based on their daily interactions with health care workers and patients as a one-page activity in their portfolios. Ethical clearance was obtained from the Human Research Ethics Committee (HREC- Medical: M110740) of the Faculty of Health Sciences at the University of the Witwatersrand. All 25 students agreed to participate in the study and submitted written informed consent for their portfolios to be included in the research. Only portfolios that reflected on time spent in three of the five rotations, namely: Paediatrics (Paeds), Emergency Medicine (EM) and Adult in-Patient wards (AIPW) formed part of the study as students rotated in sites in the Gauteng province and rural communities in the North West province. With the closet site being a mere 10 km away from medical school, and the most distant site some 450 km away, this adequately covered the BCMP scope of practice and represented the maximum spectrum of a clinical training environment [21]. Thus the portfolios reflecting on the time spent in Surgery and in the Outpatients department were excluded from this analysis as the researcher was of the view that students would have similar experiences as reported in EM and in AIPW. Also, including all five clinical departments would have extended the scope beyond the research mandate.

A retrospective design method was employed as data was obtained from portfolios that were initially designated as tools for the formative assessment of a block placement. Whilst a retrospective case design is perceived to be a quick and easy approach, its main challenge is that it tends to rely on recall. For the BCMP students, reflecting on their experiences was considered to be a critical exercise as this was used to evaluate both moral and academic functioning as a measure of professional integrity as the students personally decided on the content of their discussions [21]. Also in the context of Wits as a learning environment, written reflections in the form of portfolios are used extensively to link theoretical knowledge with clinical experiences undertaken under supervision [12]. An exploratory, descriptive approach allowed for a greater understanding of the BCMP students’ experiences considering the context, the behaviours and attitudes related to professionalism [22-24].

A total of 71 portfolios received for formative assessment as hard copies were available for analysis instead of the 75 that were expected. The other four that were submitted electronically were lost with the theft of the programme coordinator’s computer. Thus the return rate for the portfolios was 100% (n = 25) for EM only, for AIP the rate was 92% (n = 23), similar to Paeds at 92% (n = 23). Portfolios were chosen as the ideal instruments as they facilitate independent reporting by students and also allow for critical reflection and an honest discussion [24-26]. They are also considered to be effective and efficient tools of assessment in primary care [27]. The portfolios were all written in English and submitted as hard copies ranging from one to five pages dependant on whether the document was typed or handwritten. There was no limitation to the word count, a deviation from similar studies where students were given specific instructions [21]. No other socio-demographic data were available for any of the students as part of this analysis as the portfolios were anonymized to protect the identities of the students [28]. As a newly launched programme there was extensive press coverage with photos taken of the students at each briefing. As the class comprised of only 25 students, it was felt that the students might be potentially victimized with subsequent adverse outcomes on their learning experiences hence a blinded study. At the point of getting informed consent, students were informed of the intentions of the researcher to include, where it was deemed necessary direct quotes from their portfolios. As part of the data analysis process, each of the portfolios was given a unique identifier that was closely linked to the clinical department that the student was placed in at the time of their reflection. The researcher read through each of the portfolios (Paeds; EM and AIPW) to get a sense of the students’ writing styles and create data sets. To authenticate data sets, the researcher read through additional portfolios from the same discipline to ascertain if students followed and responded to the instruction in a similar manner [22]. The process of immersion was repeated many times over to validate earlier findings and find meaning in each of the stories narrated by the students. For this reason the qualitative approach was a method of choice to analyze the open-ended reflections. Content analysis was employed to examine emerging themes, meanings and patterns from the submitted portfolios within an ethical framework as described by Malpas [29] and quantify the frequency with which they were articulated using Excel as a simple descriptive tool [24]. Credibility of the data was enhanced through a variety of strategies that included description of setting, methods and triangulation whereby portfolios were collected from different training sites to represent each of the three clinical training departments. Independent confirmation of the data sets as well as the interpretation of results was supported by one of the co-authors –ER who has extensive experience in qualitative methodology.

## Results

Three themes were identified that supported the BCMP students’ reflections on the development of their personal attributes of professionalism. The three themes categorized as personal attributes comprised of accountability and responsibility, critical reflection linked to professional development, as well as personal growth. The results of each theme are presented in Table 1 below.

**Table 1:** Personal attributes of professionalism

**Personal attributes**	**BCMP students’ reflections/per department**			**Frequency of reflections in portfolios**
**Attributes of reflective practice**	**Paeds**	**AIPW**	**EM**	**Total**
	**n = 23**	**n = 23**	**n = 25**	**N = 71**
**1. Accountability and responsibility**	18	21	15	*5*4
**2. Critical reflection**	17	17	17	51
**3. Personal growth**	7	12	6	25

Key: Paeds = Paediatrics; AIPW = Adult In-Patient Ward; EM = Emergency Medicine; Health Care Professional (HCP) or Health Care Professionals (HCPs) is used interchangeably with Health Care Worker (HCW) or Health Care Workers (HCWs).

### Accountability and responsibility

In the context of this theme, the majority of BCMP students (n = 54) reflected on the determinants of accountable and responsible practice and sought to define ethical and unethical behaviour based on their experiences. As the students encountered a range of ethical issues and challenges, they referred to the requirements of standards of good practice as determined by the Oath as encapsulated below:

“When I started the BCMP we were all asked to take an Oath; this Oath meant that patients will always come first and everything else later.”

The expectation was that this knowledge would automatically transfer to patient care, with the emphasis on the need to treat all patients with empathy:

“*The Oath has no significance if HCP mistreats patients*…*We take the Oath not because we are doctors but because sooner or later we are all patients.”*

The commitment to the profession as a public duty was not lost to the BCMP students. In their reflections, a commitment to the profession guided the care that was provided to patients:

“*Until HCW realise that they are public servants, they will not fulfil their duties. Being a public servant means rendering good standard service with passion and effectiveness…”*

Some students highlighted the fact that at the point of consultation patients are vulnerable and that HCPs had a corresponding responsibility as determined by their obligation to treat patients as people:

“*To be regarded as a good HCP requires a long-life commitment and an overriding dedication to one’s fellow human beings and society.”*

Students were of the view that the interaction between HCPs and patients was a relationship founded on trust and one that required every consultation to reflect this trust. Reflecting on the covenant that exists between the profession and society, one of the students cited patients as having multiple roles in society and thus deserving of the respect that was due to them at facility level:

“*A patient is not just a patient she is a mother, a wife or a friend. As HCWs we serve, we care and save lives… Our job as HCW has to be done to perfection - there’s no room for lousy mistakes because when we make mistakes, people die and that defeats our purpose in the health system.”*

The principles of good practice were referenced a number of times in relation to patient care as prescribed by the Health Professions Council of South Africa [30] with a distinction drawn between health care providers and professional health care providers:

“*Being a health care provider is one thing but being a* ***professional health care provider*** *is something else because you need to know ethical standards very well and understand their meaning.*”

Thus the ideal HCP was perceived to be someone capable of incorporating core ethical values and standards of good practice as part of their daily interactions with patients:

“*One should bear in mind that these standards are not set just to fill an Act booklet, but they are there for us HCP to practice them*…*”*

The attitudes of HCPs were regarded highly and linked to outcomes of the consultation process. Professional HCWs were described as those HCWs whose behaviour was exemplary. A recurring observation was a reference to HCPs who showed commitment to patient care:

“Credit must be given to the minority of HCW who are still committed to their jobs such as Sr S. What I like and applaud her for is the fact that she took her own initiative and walked an extra mile to make the patient feel much better.”

Students were of the view that raging tempers and shouting at patients were all attributes that compromised patient care as one student recalled a verbal dispute witnessed between a HCP and a patient’s mother. The exchange had a far reaching impact on the student. As an outcome of this negative experience, the student reflected on the impact of this experience towards his approach to patients as follows:

“I resolved to learn to control my temper, my daily frustrations and be wary of the control that my mood might have on the outcome of the patient consultation.”

Imparting information to patients was considered by many of the students to be a critical tool in closing the knowledge gap between the professional HCP and the patient expressed as a point of concern by one of the students:

“*HCPs seem to be forgetting that patients have a right to be told about their illness and given an explanation as to why we take bloods or put up drips.”*

In addition, patient education was considered to be one factor that would limit non-compliance and unnecessary hospitalization:

“*The 12 year old’ right to refuse treatment is not informed if he is RVD reactive, defaults on treatment and refuses transfusion for fear of contamination by infected blood.*”

Death was an experience that was recognized as the necessary end but at times raised ethical dilemmas presenting either as a challenge or an opportunity. The high mortality rates were depressing to some of the students but they learned from the HCWs to move on and continue to render the required health care to patients. The students decried the lack of debriefing sessions as they struggled to deal with their own emotions:

“*When death is no stranger to your setting where should one find refuge especially when everyone has accepted this as a norm?*”

For one student, death presented an opportunity for the student to practice an important skill, namely, that of breaking bad news to families:

“I feel like being in a District Hospital is very depressing because most of the time you will watch patients dying in front of you…I witnessed death every week of the rotation about two people per week most of them were from Stage 4 HIV. I also had a chance to break bad news to the family of the deceased.”

At times lack of resources was blamed for mortality in EM and AIPW. Students understood that they could not “play God” and that it was not possible to save all the patients. However, there were instances when the students spoke of a wrongful death when patients had died due to lack of equipment as expressed below:

“Shortage of beds is not only an inconvenience to doctors but also increases the mortality…and the transmission of illness to other patients and to staff.”

There were also instances where the families of sick patients violated hospital protocol and abused state resources particularly in AIPW and EM, which were of concern to students hence indicating that the responsibility and accountability were perceived by students to apply to patients too.

### Critical reflection linked to professional development

This theme allowed students to reflect on their professional development as they rotated through the different departments. Many of the students (n = 51) were motivated to exhibit model behaviours, and recognized the value of constantly reflecting as a skill that develops over a period of time based on experiences that had a profound impact on their learning experiences, from assumptions they made as students to a realization that to err is human:

“*In medicine you should never say never and never say always as not every malnourished patient is HIV infected*…a*s a Clinical Associate student I have learnt that patients either die because of late medical intervention, natural causes… – as HCPs we are subject to mistakes, patients can die on account of our errors. We are human beings - educated and trained does not exempt us from infallibility.*”

They recognized the need to conform to the theoretical guidelines as one student acknowledged the wrongness of being influenced to short-circuit the accepted process of clerking patients. According to the student, this process tended to compromise patient care and led to the wrong conclusion, forcing the student to go back to the correct management approach. Context-specific approaches in obtaining sensitive patient information such as the patient’s HIV status were reflected on as positive and negative experiences. One student recognized that he was fortunate to have been taught by a patient in that it was one of the parents who recognized the student’s naivety and proceeded to show the student the correct way to elicit confidential information:

“I remember in the first week I asked one of the mothers if she was HIV positive and she said no. She then called me to speak privately with her and it was only then when she disclosed that she was HIV positive. I then realised my mistake immediately and never did it again.”

The general perception was that there was no limit to learning and sharing information in the clinical training environment. The benefits were not limited to the BCMP students as the registered learners:

*“Being a Teachable Learner made my interactions with my colleagues a lot better. I also taught some nurses some things that they needed to know which made work easier and more fun”*.

The concept of “*pushing the line”* (i.e. consulting as many patients as possible to address the long queues), was expressed as a concern as it compromised patient care.

“Pushing the line at the expense of the patient compromises privacy and confidentiality.”

Students also expressed reservations about their standing in view of the current crisis where there was an on-going shortage of HCWs. One of the students vocalized an underlying fear that this crisis might challenge their commitment to professionalism.

“…*I feel the medical crisis which South Africa is facing with the lack of doctors, professionalism is affected and slightly reshaped and warped as ultimately the fashionable trend is ‘****push the lines****’. Instilling genuine professionalism is going to be a struggle and we stand to watch and see how Clin Assoc will impact the health field and how long it will take for them to be engulfed by the ways of the health care system or if they’ll stand firm and hold their own new ground.*”

Students considered the harm that may come to patients when they are forced to “push the line” and work beyond their scope of practice.

“*How far out of my scope may I work knowing the care I can provide to a patient may be harmful*?”

Students reflected on the value of prioritizing patients by demonstrating compassion and putting the patient first, as encapsulated in the following response:

“*We spend a lot of time worrying about how many patients are waiting. You forget the one in front of you…we need to adopt a way of focusing on the patient, so the moment he walks in we should tell ourselves my patient is the one in front of me.*”

### Personal growth

As part of this theme, students focused on their concerns and referred to a period of uncertainty but attributed much value to their learning experience. Just over a third of the students (n =25) reflected on their role on being the first group to be admitted to the programme as Clinical Associates:

“*It all started with 25 Guinea pigs and hopeful personnel…initially we used to be very unsettled…it was not easy to infiltrate the hospitals people were confused…eventually it became enjoyable and it was a marvellous learning experience.”*

The majority of these experiences of professional growth were borne out of their experiences with admitted patients in Paeds and AIPW (n = 19). There was the struggle to fit in but a definite sense of pride in establishing an identity. The corresponding responsibility to earn the trust and respect of patients was considered a critical undertaking:

“*After introducing yourself as a Clin A student the follow up question in most cases is if you are going to be a doctor…in most cases patients’ trust depreciates once you tell them you are not going to be a doctor. Now you have to prove yourself then later on that’s when you get the compliments.”*

Commenting on the value of interpersonal relationships, they expressed the benefits of working together and functioning as part of a team. While they acknowledged their own strengths and their contribution to the welfare of patients, they firmly corrected any misperceptions on their professional status, with one student reflecting following a successful attempt to resuscitate a patient with asthma:

“…*by the time the doctor came back the patient was not in distress and all the vitals were normal and all he had to do was countersign all that I did and prescribe medication for the patient. After all this I gained respect from the sisters and they started calling me Doctor…I correct them…I am Clinical Associate not a Doctor.”*

BCMP students allowed themselves to transition from student to a health care professional in a training capacity while acknowledging the bonds that were necessary to sustain their personal development. The ultimate reflection was in regard to the clinical training environment preparing and providing insight for future practice:

“*It is amazing that we are able to write journals and we are not short of ethical issues. It is not good for patients to notice them but for us students it is a good thing to observe so we don’t make the same mistakes and these things shape us to be better professionals.”*

There was a perception of safety networks provided by the training environment, an opportunity to learn from one’s mistakes to get to the level where they needed to be:

“*I didn’t know the chest compressions were so tiring and the human chest is softer than the manikin we practise on in medical school. Two minutes into chest compression you are exhausted and I guess you need to do it more often to get better.*”

Students acknowledged the responsibility of carrying the programme and bridging the gap between public duty and societal expectations as they educated patients about the new profession:

“*As a carrier of the title, we have to understand the name so we may portray the name…If we as Clinical Assoc…and behaving in a manner that which is expected of Clinical Assoc then the public will certainly notice the difference and start questioning…with that the patients will see the difference.”*

In this study the development of professionalism as a reflection process constituted a summation of a three-year integration of theory and clinical work [24,28]. However, due to the anonymity of the data, it was not possible to share the preliminary findings of the study with the graduates. This was subsequently achieved at the end of the research process as a copy of the research report was emailed to the graduates’ personal email addresses.

## Discussion

The emotions expressed by the BCMP students both in communicating their career aspirations as well as their fears by acknowledging their limited skills as students reflected on their journey with similar enthusiasm as reported by Green-Thompson et al. [12]. Reflecting on the development of their personal attributes of professionalism, their experiences compared relatively to findings observed by senior medical students in Australia and by fifth-year medical students in a university in the US [21,29]. In this study, the most critical finding was the internalization of the Oath being directly linked to BCMP students showing empathy to their patients. This value that students assigned to the Oath is supported by Harris [31] who refers to the role of the Hippocratic Oath as an undertaking that drives the health care worker’s call to be a physician and a guide for whatever intervention is provided to patients. Branch Jr. [25] reported similar findings on critical incidents reports submitted by third-year medical students who developed an empathic relationship with their patients.

In this study, the factors that compel one to believe that the BCMP students had internalized the Oath have been their ability to link the Oath to ethical standards of good practice described by the Health Professions Council of South Africa [30] and the expected commitment for HCWs to provide the same care they would expect if they were admitted as patients. The second reason perceived to fulfil Stern and Papadakis’s [9] aspirations was facilitated through early exposure to the clinical environment and reinforced learning that was provided by an integrated curriculum. In this way, their commitment to the Oaths or expected commitment of other HCWs provided a direct link to professionalism through the three ethical principles of patient welfare, social justice and respect for patient autonomy [6,32]. Hafferty [33] offers a different view to current findings as the results of his US study present medical students as being unreceptive to the notion of Oaths as they were not able to integrate values and duty for future practice. Stern and Papadakis [9] blame this weakness on the structure of the curriculum where ethics teaching is introduced early on, with positive reinforcement only implemented in the latter part of the training period.

The student-patient interactions were reported as constructive experiences as the case of the student who reflected on being taught by the mother of a patient the proper approach to soliciting sensitive patient information. Final-year medical students both at the University of Pretoria (SA) and Wits also reported on positive experiences whilst interacting with patients [12,34]. The personalized reference to patients as “*my patient*” was evidence of a period of growth from students feeling like “*guinea pigs”* to “*teachable learners”* who accept the responsibility that embodies the profession [12]. This reflection is supported by Baingana *et al.*[5] who report on senior students’ perceptions on learning about professionalism at Makerere University in Uganda as the times when you are alone and doing the right thing, guided by the standards of good practice.

The views expressed by students in this study in regard to the interactions with other members of the health care team as positive experiences and opportunities for reflective practice simulate those reported by Bergh *et al.*[34] and supported by Irvine [35] who addressed this very topic as president of the British General Medical Council. He called for doctors to respond to patients as knowledgeable, skilled, ethical and committed HCWs. Furthermore, Irvine called for an improved relationship that responded to patients and their families, thereby ensuring that they have sufficient information, sufficient choice and sufficient autonomy. According Pellegrino [36] each patient encounter is an opportunity for every HCW to demonstrate commitment to the profession by using attained competence to demonstrate an altruistic approach towards patients. Kaldjian *et al.* and Gordon support opportunities that expose students to reflecting about ethics and the values of professionalism in clinical practice [21,37].

BCMP students’ reflections on negative experiences as instances where student learning and patient care were compromised either due to inadequate communication, lack of resources, attitudes or shortage of HCWs undermines the perception of the practice of medicine as a noble profession [35]. Other behaviours that compromised patients such as observed verbal exchange between a patient and a HCW are similar to the professional lapses reported by Vivian *et al.*[38] where students at the University of Cape Town (UCT) observed similar incidences and were not empowered enough to challenge the HCW [39]. It is perhaps the quadruple burden of disease described by Mayosi *et al.*[40] that has culminated in what has been dubbed a medical crisis in South Africa by the BCMP students as they refer to the notion of “*pushing the line*”. Mayosi *et al.* blame rapid industrialization for the rising burden of non-communicable diseases in rural areas and among poor people residing in urban communities calling for the strengthening of district health services. The concerns expressed by Mayosi *et al.* regarding the increasing demand for health services were reflected in the experiences of the BCMP students as they undertook their training in District Education Campuses. These negative findings need to be viewed against the backdrop of the current state of health care in South Africa. For example, Dhai [41] refers to the current state of health care in this country as one that can be blamed on system deficiencies, lack of accountability and declining standards of care as a result of late or non-payments of suppliers, incompetent managers and non-delivery of services.

The burden that comes with the high number of patients seeking treatment in a training hospital was observed by fifth-year medical students in Uganda [5]. However, no reference in that study undertaken by Baingana *et al.* was made to the high patient load being a burden on the students except in regard to the impact it had on resources and attitudes of staff [5]. Whilst the challenges reported by BCMP students may be viewed as negative experiences, unintended benefits were reported as instances where a negative experience such as death of patients reinforced the BCMP students’ skills development. Mueller [6] supports activities that engage students in improving their communication skills, referring to opportunities that allow students to learn about breaking bad/sad news to patients. Bergh *et al*. [34] also reported on opportunistic learning following the death of a patient when the students had to break bad news to the family. Mahood [27] refers to primary health care/community practice as the sector most vulnerable to staffing challenges and lack of resources where students are most likely to be turned into ethical chameleons. The call by Mahood for vigilance in these settings in order to safeguard the interests of students as trainees is one that should be observed.

### Limitations

The nature of the study resulted in methodological limitations as the research material was a task intended primarily for formative assessment of the rotations. As this was a blinded study, it has not been possible to provide a true identity of those who had the most positive attributes nor was it possible to identify the facilities where the BCMP students had the most positive learning experiences. As the study was based on only one cohort of BCMP students, this may preclude generalization of findings to other groups of BCMP students trained at other universities.

## Conclusion

The discussion that constituted the development of personal attributes of professionalism was a reflection of how the BCMP students perceived their formal curriculum relevant to their lived experiences during clinical rotations in Paeds, EM and AIPW. Through their portfolio narratives, BCMP students showed a willingness to shape their evolving journeys of moral growth and personal development. They also exhibited an extended commitment to their profession and a commitment to take on the role of future role models. Similarly, the District Education Campuses as decentralized training platforms provided the context where students had opportunities to learn from professional HCWs and patients. This study has highlighted as an ongoing challenge the need to identify a process by which professionalism is sustained by HCWs to benefit health sciences students.

## Competing interests

The authors declare that they have no competing interests.

## Authors’ contributions

NM-S conceptualized the research and drafted the article. AD and NT contributed to the conception, the design and empirical analysis of the study. ER provided expert advice in qualitative research methods. All three co-authors made substantial contributions during the writing phase of the study and also read and approved the final manuscript.

## Pre-publication history

The pre-publication history for this paper can be accessed here:

http://www.biomedcentral.com/1472-6920/14/146/prepub

## Supplementary Material

Additional file 1 BCMP Curriculum Overview. Click here for file
