# Supplementary material for: Developing personal attributes of professionalism during clinical rotations: views of final year bachelor of clinical medical practice students
Source: BMC Med Educ. 2014 Jul 16;14:146. doi: 10.1186/1472-6920-14-146 (PMC4107478; doi:10.1186/1472-6920-14-146)
Supplement: Additional file 1 — BCMP Curriculum Overview. [file 1472-6920-14-146-S1.pdf]

Additional file 1: BCMP Curriculum Overview

Appendix A: BCMP Year 1, 2 & 3 Curriculum Overview: Theory and Clinical Areas

YEAR 1	Block 1	Block 2	Block 3	Block 4
Weeks	7 weeks	10 weeks	7 weeks	8 weeks
Theory	Medical Biology, Microbiology & Biochemistry	Respiratory System	Gastrointestinal System	Renal System
	Systems Overview -Anatomy & Physiology	Cardiovascular System	Musculoskeletal System	Reproductive System
	Defenses: Immune System	(Integrated approach)	(Integrated approach)	Neurologic System
	Homeostasis: Endocrine System			Special Senses
	Pharmacology			(Integrated approach)
Skills Lab	½ day per week	½ day per week	½ day per week	½ day per week
Anatomy Lab	½ day per month	½ day per month	½ day per month	½ day per month
Clinical Areas	Wards (1 full day/wk)	Wards (1 full day/wk)	Wards (1 full day/wk)	Wards (1 full day/wk)
		OPD (1/2 day/wk)	OPD (1/2 day/wk)	OPD (1/2 day/wk)

YEAR 2 Weeks	**Block 1**- TBL: Adult Health, 9 weeks	**Block 2**- Clinical Experience: Adult Health, 12 weeks	**Block 3**-TBL: Women, Child, Adoles Health 6 weeks	**Block 4**- Clinical Experience: Women, Child Health, 9 wks
	Blood: Anaemia & Malaria			
	Liver Disease (Pharm Rev)	OPD (Daily for 4 wks)	Women's Health	Antenatal
	HIV & TB (Immune Rev)	HIV (Daily for 4 wks)	Gynecological & OB	Maternity
	Emergency Trauma	Wards (Daily for 4 wks)	Embryology	Postnatal
	Neurologic Conditions	After hrs (Casualty15hrs/wk)	Neonatal Health	Family Planning
	Opthalmologic Conditions	TBL (1 time wkly)	Child Health	Paeds (3 wks)
	ENT Conditions	PBL (1 time wkly)	IMCI	Casualty (3 wks)
	Respiratory Conditions	CME (1 time wkly)	Adolescent Health	Afterhrs (Casualty 15hrs/wk)
	CVS Conditions			TBL (1 time wkly)
	Endocrine Conditions			PBL (1 time wkly)
	Renal Conditions			CME (1 time wkly)
	GI Conditions			
	Muscu-skeletal Conditions			
	Dermatologic Conditions			
	Men's Health			
	Occupational Health			
	Geriatric Health			
Skills Lab	½ day week			
Anatomy Lab	TBA			
Clinic Area	OPD ½ day per wk			

YEAR 3	**Block 1**	**Block 2**		**Block 3**	
TBL #1	Rotation 1 & 2	TBL #2	Rotation 3 & 4	Rotation 5	Rotation 6
3 weeks	5 weeks each	3 weeks	5 weeks each	5 weeks	5 weeks
Emergency Medicine Workshop	Emergency Rotation	Research Project-Protocol writing	Emergency Rotation	Emergency Rotation	Elective
Research Project-Intro to Protocol	Surgical Rotation	Emergency Med, ACLS Review	Surgical Rotation	Surgical Rotation	*(OB/GYN, Orthopedics,*
Surgery Overview	Paediatric Rotation		Paediatric Rotation	Paediatric Rotation	*Trauma,*
In-Pt, Out-Pt & Paediatrics Review	In-Pt Wards & Out-Pt Clinic Rotation		In-Pt Wards & Out-Pt Clinic Rotation	In-Pt Wards & Out-Pt Clinic Rotation	*Paeds, HIV, etc)*
Mental Health	Mental Health/HIV Rotation		Mental Health/HIV Rotation	Mental Health/HIV Rotation	
OPD (1/2 day/wk)	Afterhours (15hrs/wk)	OPD (1/2 day/wk)	Afterhours (15hrs/wk)	Afterhours (15hrs/wk)	Afterhours (15hrs/wk)
